# Supplementary material for: The Generation of an Artificial ATP Deficit Triggers Antibiotic Production in Streptomyces lividans
Source: Antibiotics (Basel). 2022 Aug 27;11(9):1157. doi: 10.3390/antibiotics11091157 (PMC9495134; doi:10.3390/antibiotics11091157)
Supplement: Supplementary file 1 [file antibiotics-11-01157-s001.zip › antibiotics-1851968-supplementary.pdf]

**Supplementary Materials**:

**Table S1** Oligonucleotides used in this study

| Purpose | Sequence |
|---|---|
| **Internal deletion of 36bp in *atpD*** | |
| NS1 | 5'-GATCGGTCTGCAGGAGATGATCTACCGCGT-3' |
| NS2 | 3'-TCCCGCCGTTCTAGCCAGACGTCCTCTACT-5' |
| **Primers for RT-PCR (Figure 3)** | |
| *HrdB* forward | 5'-AAGGAAGACGGCGAGCTTCT-3' |
| *HrdB* reverse | 5'-GCACCGGGATACGGATGGTG-3' |
| *CpkA* forward | 5'-AGGTCGGTCTGGTCGAGTTGT-3' |
| *CpkA* reverse | 5'-CGCACAAGAACTCCAGCATGA-3' |
| *CdaR* forward | 5'-ACTCGCTCCGTTTCGAACAACT-3' |
| *CdaR* reverse | 5'-GAGGTCGGTACCCAGTTCAAGA-3' |
| *Cda* PSI forward | 5'-GGCTTCCTCTTCCTCAACCTGT-3' |
| *Cda* PSI reverse | 5'-ACGTACAGCTCGGTCAGTTCCT-3' |
| *RedD* forward | 5'-CAACATATTGGGACCCGTATCG-3' |
| *RedD* reverse | 5'-ATCGCGCTGATGAGATCCTC-3' |
| *RedL* forward | 5'-AGCCGAGATAGCACGGTTCATC-3' |
| *RedL* reverse | 5'-AGATGCCGGTGGAGAAGATGTG-3' |
| *ActII ORF4* forward | 5'-GTGTCCATGTAATCACCGATGC-3' |
| *ActII ORF4* reverse | 5'-AATTACCAGGGACCGGAGTTCC-3' |
| *ActII ORF1* forward | 5'-TGAAGCGCAGAGTCGTCATCAC-3' |
| *ActII ORF1* reverse | 5'-ACCAGGTAGTCGAACATGTGCC-3 |

**Primer PCR (Figure S1)**

Within promoter *ErmE** upstream of *atpA*

5'-GACGGT*ATCGAT*AAGCTTGC-3'

*atpA* reverse

5'–TCCTCGATGCCGCTGAACT -3'

**Primers for RT-PCR (Figure S2)**

| | |
|---|---|
| NRPS/SCO6431 forward | 5'-GGCGTTCATCTTCTACACCTCC-3' |
| NRPS/SCO6431 reverse | 5'-AGTCCGTAGACGTTGAACAGCC-3' |
| EizA/SCO5222 forward | 5'-ATATGCCGACGGTCTGTGCTAC-3' |
| EizA/SCO5222 reverse | 5'-TGAGCAGAGGTCGTTGTACCAG-3' |
| CpkA/SCO6275 forward | 5'-AGGTCGGTCTGGTCGAGTTGT-3' |
| CpkA/SCO6275 reverse | 5'-CGCACAAGAACTCCAGCATGA-3' |
| CrtE/SCO0185 forward | 5'-ACCTTGAAGAGCGCCCTGTA-3' |
| CrtE/SCO0185 reverse | 5'-AGGCTCATGTCCGTCAGCTC-3' |
| CchH/SCO0492 forward | 5'-CAGCTTCTCCACCTGACGGTAA-3' |
| CchH/SCO0492 reverse | 5'-CGCTTCTCTCGTACGGCTTTCT -3' |

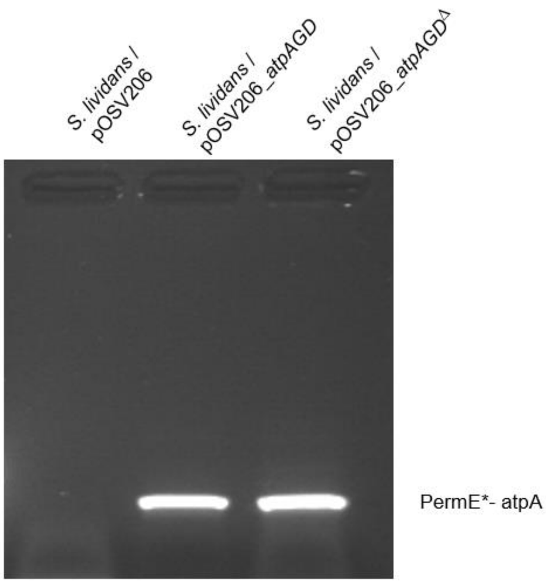


**Figure S1**: Determination by RT-PCR of the level of transcription of the *atpAGD* cluster present on the plasmid pOSV260 in S. lividans/pOSV206_*atpAGD* and S. lividans/pOSV206_*atpAGD*$^{\Delta}$, grown on solid HT medium for 48 h, with specific primers designed to amplify specifically the copy of the *atpAGD* cluster carried by the plasmid and not the genomic copy of this cluster (Table S1).

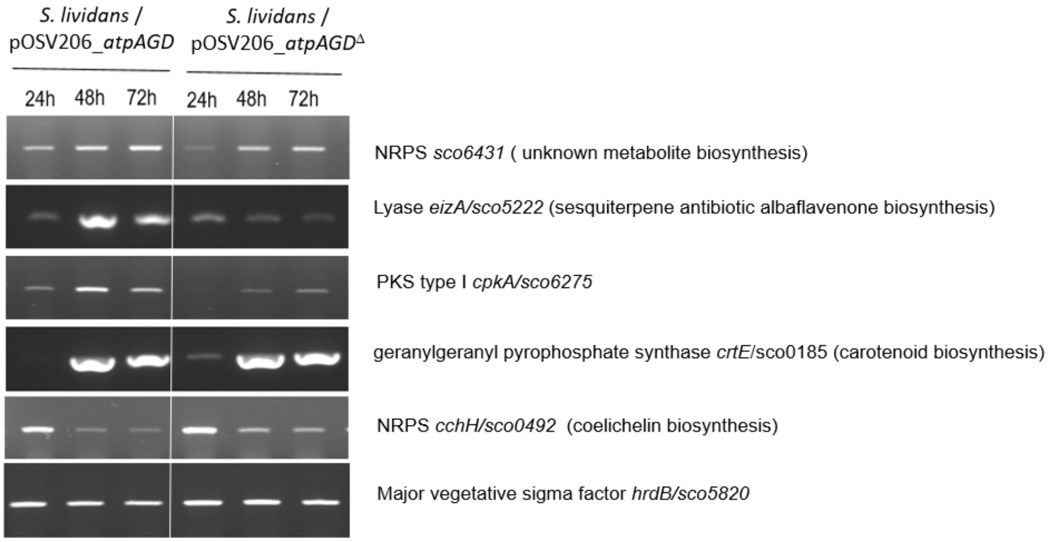


**Figure S2**: Determination by RT-PCR of the level of expression of genes belonging to various specialized metabolite biosynthetic pathways in S. lividans/pOSV206_*atpAGD* and S. lividans/pOSV206_*atpAGD*$^{\Delta}$, grown on solid HT medium for 48 h, using the primers specific to each gene listed in Table S1. These include the NRPS *sco6431*, *eizA*/*sco5222* involved in the biosynthesis of albaflavenone, *cpkA*/*sco6275* encoding a PKS of type I involved in the biosynthesis of a cryptic yellow polyketide, *crtE*/*sco0185* involved in carotenoid biosynthesis and that of the NRPS *cchH*/*sco0492* involved in coelichelin biosynthesis. *hrdB*/*sco5820* PCR amplification was used as internal control.
